# Yellow Fever

**Published:** 1898-04-23

**Authors:** 


### YELLOW FEVER.

Yellow fever has for so long made itself a home in Cuba, and has caused such losses to the Spanish troops employed there, that one of the troubles to be reckoned with by the United States, should intervention in the affairs of that unhappy island be undertaken, is the chance of a large recrudescence of this most fatal disease. Wars always stir up latent epidemics, and often increase their virulence to an extraordinary extent. Great interest, then, attaches to the recent investigations of Dr. Sanarelli into the bacteriology of yellow fever, and to his attempts to obtain an antidote. The serum, which is now being used by Dr. Sanarelli in the treatment of yellow fever, is obtained from horses who have been rendered immune by prolonged intensive treatment carried out for a year or more, much on the same lines as in the production of anti-diphtheritic serum. Great difficulty has, however, been experienced in obtaining a product of sufficient power, owing to the fact that animals cannot readily be made to tolerate strong doses of the virus. The serum so far obtained, although it is "anti" to the microbe, is not an antitoxin in the true sense of the word—*i.e.*, it does not appear to neutralize toxins which have already been produced. Hence, it is efficacious only when used in the early stage of the diseases, before the toxins have profoundly affected the kidneys and the central nervous system. The results obtained have been moderately successful, as, in fact, was as much as was to be expected. In estimating the action of such a remedy, we must compare it with others of the same class. Even the anti-diphtheritic serum, the very type of this class of drug, is by no means a certain cure when the disease has once been allowed to make headway, notwithstanding its striking efficacy when used sufficiently early. So far as we can judge from the small number of cases reported, the use of the serum appears to have been attended with a lowered mortality.

